# Adverse drug reaction reporting by community pharmacists in the Greater Accra Region of Ghana, 2016

**DOI:** 10.4314/gmj.v55i1.3

**Published:** 2021-03

**Authors:** Johnson Y Osei, Priscillia A Nortey, Delia A Bandoh, Ernest Kenu, Adolphina A Addo-Lartey

**Affiliations:** 1 Department of Epidemiology and Disease Control, School of Public Health, University of Ghana, Legon. P. O. Box LG 13, Accra, Ghana; 2 Ghana Field Epidemiology and laboratory Programme, School of Public Health, College of Health Sciences, University of Ghana, Legon. P. O. Box LG 13, Accra, Ghana

**Keywords:** Pharmacovigilance, Adverse drug reaction, Pharmacists, Ghana

## Abstract

**Objectives:**

To assess adverse drug reactions (ADRs) reporting and identify factors to improve ADR reporting among community pharmacists in the Greater Accra Region of Ghana.

**Design:**

A quantitative cross-sectional study.

**Setting:**

Community pharmacies in the Greater Accra Region of Ghana.

**Participants:**

We randomly selected 210 pharmacists from a list community of pharmacies in Accra, Ghana. All participants had been practicing in the past one year, with this study being conducted from June to July 2016.

**Main outcome measure:**

Prevalence of ADR reporting by community pharmacists in Accra, Ghana.

**Results:**

Of the 210 community pharmacists interviewed 54.0% were males. Mean age was 32±10 years. Majority (96.0%) had heard of ADR reporting in Ghana, yet 18% had never seen the ADR reporting form. Reasons given for failure to report suspected ADRs included unavailability of reporting forms (83.1%), uncertainty about a causal relationship between the drug and the suspected ADR and classification of the reaction as “normal” with the medication being taken (23.6%). Only 34.0% of pharmacists had the ADR reporting forms available in their facilities. Marital status was the only factor significantly associated with ADR reporting (OR 3.18, 95%CI 1.02 – 9.12).

**Conclusion:**

ADR reporting by community pharmacists in Ghana remains low. To improve the proportion of reporting, ADR forms should be made available in all pharmacies, pharmacists and the general public should be made aware of online reporting systems, with continuous professional development in Pharmacovigilance with the advice that all suspected ADRs should be reported irrespective of uncertainty about causality.

**Funding:**

None declared

## Introduction

One of the key goals in pharmacotherapy is to optimize patient care by increasing drug efficacy and reducing drug toxicity. However, this desired result is not always achieved particularly in instances where a patient suffers an adverse drug reaction (ADR). An ADR is defined as a response to a drug which is noxious and unintended, and which occurs at doses normally used in man for the prophylaxis, diagnosis, or therapy of disease, or the modifications of physiological function.^1^ ADRs are a leading cause of mortality and morbidity globally.^2^ In Europe, 20% of ambulatory patients on drug therapy experience ADRs while drug-related problems account for 10 – 20% of geriatric hospital admissions.^3^ It is therefore imperative that strong medication monitoring systems are put in place to protect patients from the harmful effects of medications.

Pharmacovigilance is defined as, “The science and activities relating to the detection, assessment, understanding, and prevention of adverse effects or any other drug-related problems”.^1^ Reporting of ADRs constitutes an integral part of the pharmacovigilance process. Spontaneous Reporting Systems (SRSs) are the commonest means of reporting suspected ADRs in most countries.^4^ However, underreporting remains a challenge even in developed countries.

The global database of individual case safety reports (VigiBase) is managed by the WHO Collaborating Centre for International Drug Monitoring (Uppsala Monitoring Centre), in Sweden. It has been estimated that only 6%–10% of all ADRs are reported to this global database.^5^

Some common reasons for underreporting of ADRs include the absence of reporting forms, uncertainty about the causal relationship between the drug and ADR and perception that the ADR is “normal”^6^,^7^,^8^

According to Isah et al (2012), in Africa, self-medication is rampant with easy access to both over the counter (OTC) medications and prescription-only medicines (POM) in most community pharmacies.^6^ Irrational medicine use is rife among both healthcare providers and consumers further increasing the risk of drug-related harm. Polypharmacy, inappropriate pharmaceutical promotional activities, and irrational prescribing are also common in many parts of Africa.^6^ Community pharmacists could play an important role in the detection, documentation, and prevention of ADRs because of the large numbers of patients they see.^9^ However, there is a paucity of information in Ghana among this group of healthcare providers in assessing ADR reporting rates. This study, therefore, investigated the ADR reporting and the factors influencing the reporting of ADRs by community pharmacists in the Greater Accra region of Ghana.

## Methods

### Study Design

This study was a cross-sectional survey of ADR reporting by community pharmacists within the Greater Accra Region from June to July 2016. Pharmacists were selected using a computer-assisted random sampling of community pharmacies where they worked, and data was collected using self-administered questionnaires at the various pharmacies.

### Study Setting and Population

The Greater Accra region was chosen as the study setting because it has the largest number of registered community pharmacies in Ghana; approximately 1,048 according to the Pharmacy Council of Ghana.

### Sample size and sampling

The sample size was calculated using Cochran's sample size formula. 10 The prevalence of ADR reporting among physicians in the Greater Accra Region of Ghana as reported by Sabblah *et al* (2014) was 20%.^8^ Hence assuming expected prevalence among pharmacists will be the same as in physicians 20% (i.e., 0.2) and Q is 0.8. of ADR reporting in the region among community pharmacists, with precision, d of 0.05 at 95% confidence interval (Z-value of 1.96), the calculated sample size was 246. Using Cochran's correction formula, when population < 50,000 the minimum sample size for this study was determined as 200, however, this was increased by 25% to cater for non-response of participants. The estimated sample size was then rounded off to 250 pharmacists.

The community pharmacies were selected via simple random sampling. A list of 1,048 pharmacies representing all registered community pharmacies in the Greater Accra was obtained from the Pharmacy Council of Ghana. These pharmacies were arranged in alphabetical order and duly numbered in the Pharmacy Council database. A set of 250 random numbers between 1 and 1,048 was generated using Microsoft Excel computer software, Microsoft Office 2010. Pharmacies whose numbers were generated by the software out of the total 1,048 were included in the study.

Although some pharmacies employ more than one pharmacist, in each selected pharmacy, only one pharmacist works per shift. Thus, one pharmacist was sampled per pharmacy and there was no need for random sampling via balloting in each pharmacy.

The study questionnaire was pretested in the Ashanti Region to determine its suitability for this study since that region has the second-largest concentration of pharmacies and pharmacists after the Greater Accra per the Pharmacy Council. Ten of the questionnaires were pretested. Pretesting resulted in a few revisions, notably rewording of various sections of the questionnaire to improve understandability.

### Data collection

Data was collected using a semi-structured self-administered questionnaire at the various pharmacies where participating pharmacists worked. The questionnaire covered demographic information, knowledge about the ADR reporting process, factors influencing ADR reporting, and ways of improving ADR reporting. The pharmacists were encouraged to complete the questionnaires right away for collection; however, those who could not fill the questionnaire immediately were granted a maximum of three days within which to complete it. Where the pharmacist was absent, either because they were out making purchases or on lunch break, the questionnaire was left with the pharmacist assistant present to be given to the pharmacist (superintendent pharmacist if more than one works there) to fill and picked up later upon completion.

### Data Analysis

Data were analyzed using Stata Statistical Software Version 13 (College Station, TX: StataCorp LP. 2013). The proportion of community pharmacists reporting ADRs was obtained by dividing the number of community pharmacists who had seen an ADR within the past year and reported by the number of community pharmacists who had seen an ADR within the past year. The factors affecting ADR reporting among community pharmacists were assessed based on multiple responses to questions about the availability of reporting forms, time constraints, etc. Measures that could improve reporting rates were also assessed in the same manner through multiple responses to questions like the desire for incentives, simplification of reporting forms and procedure, etc. Demographic information was described using descriptive statistics (frequency tables, and bar graphs). The association between ADR reporting and the primary covariates (demographic factors, number of patients seen per day, knowledge about ADR reporting, training on ADR reporting) was tested using the Chi-square test. Logistic regression was then used to test the strength of association between ADR reporting and the independent variables (marital status, knowledge about ADR reporting, and training on ADR reporting) which had shown statistical significance under the Chi-square test. The results were expressed as odds ratios and confidence intervals with p values. A p-value <0.05 was considered statistically significant. Confounders such as age and sex were adjusted for in the multiple logistic regressions.

### Ethical Approval

Ethical approval was granted by the Ghana Health Service Ethics Review Committee, Research & Development Division, Accra Ghana (Protocol ID NO: GHSERC 84/12/15). Permission was also obtained from the managers/owners of the various pharmacies in instances where the pharmacist at the post was not the manager/owner before delivery of the questionnaires to the community pharmacists.

This was done by explaining the purpose of the study to pharmacy managers. Informed consent was obtained from all community pharmacists before they participated in the study. The study was carefully explained, and assurance given them about the anonymity and confidentiality of the information gathered.

## Results

### Background characteristics and ADR reporting by community pharmacists in Accra

A total of 210 completed questionnaires were returned out of 250 administered, giving the study a response rate of 84%. In all, 75 of the pharmacies were sampled from the Accra Metropolis, with 35 from the Tema Metropolis. Fifty of the pharmacies were located in the Ga East, Ga Central, and Adenta Municipal Districts. The rest were from Ga South, Ledzokuku-Krowor, and Ashaiman Municipal Districts. The sampled pharmacies had 54.0% male pharmacists and 46.0% female pharmacists, (Table 1). The mean age of participating pharmacists was 32 ± 10 years (ranging from 23 to 71 years). The median practice years was 2 years (ranging from 1 year to 40 years). The majority (96.0%) of respondents were Christian. About 37.0% of the respondents were married while 62.0% were single (Table 1).

**Table 1:** Background characteristics of pharmacists (N=210)

Variable	Number (%)
**Sex**	
**Male**	114 (54)
**Female**	96 (46)
**Age (years)**	
**<30**	127 (60)
**30–39**	43 (21)
**40–49**	21 (10)
**50–59**	12 (6)
**60+**	7 (3)
**Religion**	
**Christian**	202 (96)
**Muslim**	8 (4)
**Marital Status**	
**Single**	78 (37)
**Married**	131 (62)
**Divorced**	1 (1)
**Years of practice**	
**>2 years**	98 (47)
**1–2years**	112 (53)
**Training on ADR reporting**	
**Trained**	139 (66)
**Untrained**	71 (34)
**Number of patients seen per day**	
**≤50**	133 (63)
**>50**	77 (37)

The average number of patients seen per day by each pharmacist was approximately 60 patients. The respondents had practiced for an average of six years.

### Adverse drug reaction reporting by Community Pharmacists in Accra

Among the 210 respondents, 66.2% had received training on Pharmacovigilance and ADR reporting. Almost half (92/210) of the pharmacists had seen a patient with an ADR within the past one year, however, only fifteen of them had reported it by filling an ADR form. The period prevalence (June 2015 – May 2016) of ADR reporting among community pharmacists in the Greater Accra region was therefore 16.0% (15/92) in the present study. Of the fifteen pharmacists who had reported the suspected ADR in the past one year, the majority, 86.7% (13/15) had received training and education on Pharmacovigilance and ADR reporting.

**Figure 1:**
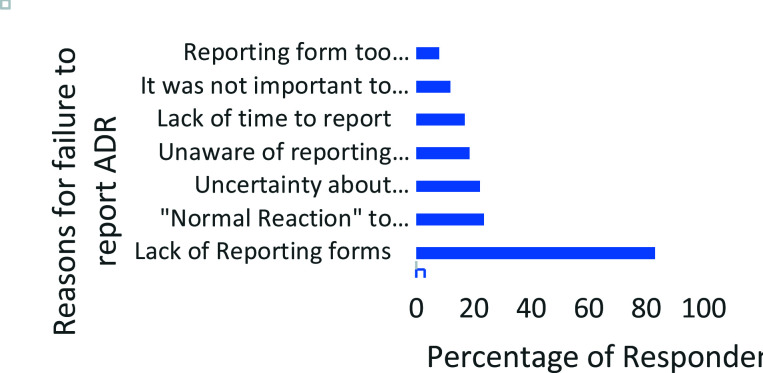
Reasons for failure to report Adverse Drug Reactions by community pharmacists.

From Figure 1, the main reason given by the pharmacists who failed to report suspected ADRs was the lack of reporting forms 83.1% (162/195). Only 36.4% (71/195) of all respondents who failed to report ADRs in the study had the reporting forms available in their facilities. Uncertainty about the causal relationship between the drug and the suspected ADR, 22.1%(43/195), and the fact that the pharmacists considered the reaction as “normal” with the medication in question 23.6% (46/195) were the other leading reasons given for non-reporting of ADR.

### Perceived factors contributing to underreporting of ADRs

About two-thirds (139/210) of the pharmacists were of the view that the unavailability of the reporting forms was a contributing factor as to why ADRs were underreported in general (Table 2). Other reasons shared were ignorance about the reporting procedures and how to obtain ADR forms 49.5% (104/210) and heavy workload and lack of time to complete the ADR forms 42.9% (90/210). Lack of confidence in the reporting system was another view that came to the forefront.

**Table 2:** Factors perceived to contribute to underreporting of adverse drug reactions

Factors	Frequency (Percent)
**Unavailability of reporting forms**	138 (65.7)
**Ignorance of reporting procedure and how to obtain** **forms**	104 (49.5)
**Heavy workload and lack of time**	90 (42.9)
**Ignorance about the need to report ADRs**	76 (36.2)
**Lack of confidence in the reporting system**	66 (31.4)
**Uncertainty about causality**	54 (25.7)
**Fear of legal consequences**	52 (24.8)
**Fear of negative publicity for my pharmacy**	51 (24.3)
**Lack of reward for reporting ADRs**	43 (20.5)
**Consumers who suffer an ADR should be responsible** **for reporting**	28 (13.3)
**Fear of negative impact on the drug manufacturer**	18 (8.6)
**All ADRs are documented before allowed onto the** **market**	13 (6.2)
**Others**	9 (4.3)
**It is not part of my professional responsibility to report** **ADRs**	2 (1)

Close to one-third (65/210) of respondents disclosed that they were not certain the ADR reports they submitted would be thoroughly investigated by the National Pharmacovigilance Centre for necessary regulatory actions. Some pharmacists provided additional reasons for not reporting ADRs, including insufficient patient awareness about reporting of ADRs 36.2% (76/210). This consequently prevented patients from reporting ADRs to their pharmacists for onward submission to the Food and Drugs Authority (FDA) (Table 2).

### Strategies to improve ADR reporting

**Figure 2:**
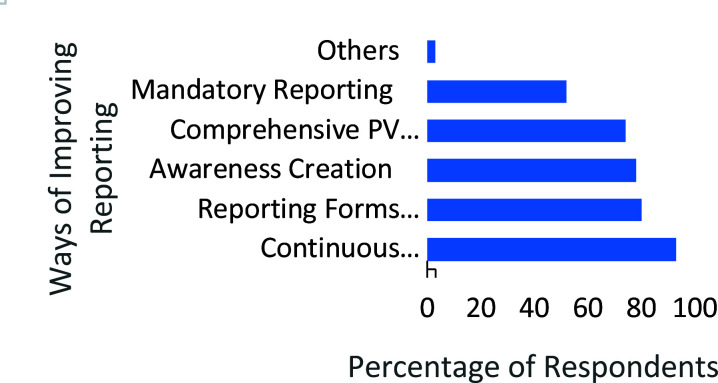
Suggested Methods of Improving ADR reporting (N=210)

About 93.0% (195/210) of the pharmacists agreed that continuous education and training on pharmacovigilance could improve ADR reporting (Figure 2). Many (168/210) of the pharmacists thought that making the reporting forms readily available in all community pharmacies would improve ADR reporting. Others suggested awareness creation among patients 78.0% (164/210) and making ADR reporting mandatory 52.0% (109/210).

Almost all the participating pharmacists (208/210) thought it was part of their professional responsibility to report ADRs. Many pharmacists also felt that doctors, nurses and medical assistants also ought to report ADRs with about 80.0% (168/210) agreeing that doctors ought to report suspected ADRs; 64.0% (134/210) stating that nurses should report ADRs and 61.0% (128/210) were of the view that medical assistants should also report ADRs.

Results for tests of associations (Table 3) showed that marital status was significantly associated with ADR reporting, with the odds of singles reporting an ADR being 3 compared to married pharmacists (X^2^= 4.23, p-value=0.04). In the multiple logistic regression factors such as marital status, training, and knowledge in ADR reporting turned out not to be statistically significant determinants of ADR reporting (Table 3).

**Table 3:** Association between background characteristics and ADR Reporting (N=210)

Variable	Frequency	X2(df), p-value	Crude OR, CI 95%	Adjusted OR, CI 95%§
**Sex**				
**Male**	114		1	
**Female**	96	0.48(1), 0.490	0.68, 0.22 – 2.06	
**Age**				
**<30**	127		1	
**30–39**	43		4.35, 0.52 – 36.28	
**40–49**	21	5.46(4), 0.243	0.36 , 0.74 – 1.77	
**50–59**	12		0.87, 0.88 – 8.63	
**60+**	7		1	
**Marital Status**				
**Married**	79		1	1
**Single**	131	4.23(1), 0.04	3.18, 1.02 – 9.12	2.28 0.66 – 7.89
**Years of practice**				
**>2 years**	98		1	
**1–2years**	112	0.64(1), 0.423	1.58, 0.51 – 4.86	
**Training on ADR reporting**				
**Trained**	139	4.16(1), 0.041	0.22, 0.41 – 1.04	2.18 0.39 – 12.28
**Untrained**	71		1	1
**Number of patients seen per day**				
**≤50**	133	0.01(1), 0.911	0.93, 0.03 – 2.90	
**>50**	77		1	
**Knowledge**				
**None / Poor**	25		1	
**Average**	28		2.93, 0.34 – 25.21	2.00 0.20 – 19.67
**Good/ Excellent**	157	8.27(4), 0.082	1	1

§ Multiple logistic regression adjusted for sex, age, years of practice and number of patients seen per day

## Discussion

Our study investigated the factors influencing the reporting of ADRs by community pharmacists in the Greater Accra region of Ghana. The study found that reporting prevalence of ADRs among community pharmacists in the Greater Accra Region was 16.0%. This figure was slightly lower than the 20.0% among doctors in the region as reported by Sabblah, et al.^11^ This was however much higher than the reported 2.9% among doctors in Nigeria.^12^ Other studies have reported similarly low reporting figures.^7^,^13^,^14^,^15^. While this study did not access how many of the patients seen had medication side effects, it is possible that patients' ignorance about their role in reporting adverse drug reactions they suffer to their community pharmacist may have played a role in the low reporting of ADRs found among pharmacists in the Greater Accra region. However, we do not have data regarding whether pharmacists were actively asking the patients about the side effects of their medications.

Thus, any inferences or interpretations of the prevalence of ADR reporting from this study must always be placed in perspective. A study among HIV patients in Ghana found that HIV patients are well informed about the adverse events of their medications and majority of them, 80.0% know they ought to report adverse events o their healthcare provider.^16^ From our findings, the recent campaign by the Food and Drugs Authority (FDA) of Ghana to increase public awareness about ADR reporting can, therefore, be viewed as a step in the right direction to tackle low reporting of ADR in the country. It launched the Patient Engagement in Medicine Safety programme in June 2016 to enable patients to report adverse events of their medicines through community pharmacies designated as Patient Safety Centres.

In the present study, there were no significant differences in age and years of practice between pharmacists who reported ADRs and non-reporters. This was consistent with findings from other studies conducted among Portuguese pharmacists which found no statistically significant association between age as well as years of practice and ADR reporting.^6^ This was also true for pharmacists in Qatar.^17^

Community pharmacists who failed to report ADRs they had encountered mostly blamed it on a lack of reporting forms, considering the reaction as “normal” and uncertainty about the causal relationship between the drug and the suspected ADR. Though the FDA had instituted an electronic system of reporting ADR alongside the option of reporting via ADR forms (paper copy), the electronic system of reporting had not yet gained widespread prominence at the time this study was conducted, hence our focus on the paper form of reporting. Nonetheless, there was an opportunity to respond other than or in addition to what was pre-coded in the questionnaire. Pharmacists, through Pharmacovigilance workshops had also been made aware by the FDA that they do not necessarily have to establish a causal link between a suspected ADR and a drug before going ahead to report it.

According to Prakasam and colleagues, most community pharmacists in India felt that the reaction they had encountered was simple and non-serious, hence their failure to report^7^. There were similar findings among 1,001 community pharmacists in Korea with the main barriers to ADR reporting being the perception of ADR being “unserious” (779/1001) and already “well known” (815/1001), as well as uncertainty about causality (733/1001).^8^

About 87% of pharmacists who had reported an ADR within the past year had received previous training on Pharmacovigilance and ADR reporting. However, training received was not significantly associated with the reporting of ADRs when factors such as knowledge and marital status were adjusted for (AOR=2.18, 95% CI: p=0.378). Similarly, there was no significant association between Portuguese pharmacists who reported adverse reactions and those who did not concerning training received in Pharmacovigilance (X^2^=3.5, p= 0.062).^6^

In contrast, training significantly improved the ADR reporting rate among medical doctors in the Greater Accra Region of Ghana (X^2^=11.6, p <0.001).(11). To improve ADR reporting by pharmacists and ADR reporting systems, new educational measures such as hands-on involvement with real cases have been suggested, thus placing ADR reporting closer to the daily routine activities of community pharmacists.^6^

The leading factors perceived by pharmacists in the study as contributing to underreporting of ADRs were the unavailability of the reporting forms and ignorance about the reporting procedure and where to obtain ADR reporting forms. This observation is consistent with findings among 310 health professionals in Saudi Arabia where 67.0% of respondents believed the ADR reporting forms were not widely available; 65.0% of the respondents felt that insufficient clinical knowledge contributed to underreporting while 63.0% blamed a lack of knowledge about the reporting address.^18^ Lack of awareness on how to report ADRs and concerns that the report submitted may be wrong were the two prominent perceived factors leading to underreporting among community pharmacists in Oman.^19^

By way of motivation, a little less than one-half of all the pharmacists in the current survey felt that a letter of acknowledgment per the report submitted was an adequate means of encouraging reporting of adverse drug reactions by community pharmacists. A little more than half of the respondents held the view that making ADR reporting mandatory in Ghana will improve reporting rates.

Likewise, more than one-half of health professionals in Saudi Arabia stated that reporting of adverse drug reactions ought to be compulsory.^18^ In Cyprus, an even greater proportion of pharmacists supported mandatory ADR reporting.^15^

Most pharmacists felt that continuous professional development and training on pharmacovigilance was an effective strategy to improve ADR reporting. This is consistent with the results of a Ugandan study where 667 out of 1589 healthcare professionals advocated for sensitization, training, and continuous education on pharmacovigilance.^20^ Moreover, about 90.0% of pharmacists in Cyprus also suggested that training in ADR reporting will help to improve the reporting rate.^15^

### Strengths and Limitations of the Study

An important limitation of this study is the issue of recall bias in that some of the community pharmacists could not accurately remember if they had encountered any ADRs. Recall on ADR reporting was therefore limited to those encountered in the past year to reduce recall bias. It is also entirely possible that some respondents might have given “inaccurate” reporting information to look good to the research or be perceived as being professional. To reduce this limitation, pharmacists were assured that they will not be penalized in any way if they give answers that might paint them in a negative light. The study also didn't specifically consider online reporting hence findings might not accurately depict the reporting rate.

## Discussion

Our study investigated the factors influencing the reporting of ADRs by community pharmacists in the Greater Accra region of Ghana. The study found that reporting prevalence of ADRs among community pharmacists in the Greater Accra Region was 16.0%. This figure was slightly lower than the 20.0% among doctors in the region as reported by Sabblah, et al.^11^

This was however much higher than the reported 2.9% among doctors in Nigeria.^12^ Other studies have reported similarly low reporting figures.^7^,^13^,^14^,^15^. While this study did not access how many of the patients seen had medication side effects, it is possible that patients' ignorance about their role in reporting adverse drug reactions they suffer to their community pharmacist may have played a role in the low reporting of ADRs found among pharmacists in the Greater Accra region. However, we do not have data regarding whether pharmacists were actively asking the patients about the side effects of their medications. Thus, any inferences or interpretations of the prevalence of ADR reporting from this study must always be placed in perspective.

A study among HIV patients in Ghana found that HIV patients are well informed about the adverse events of their medications and majority of them, 80.0% know they ought to report adverse events o their healthcare provider.^16^

From our findings above, the recent campaign by the Food and Drugs Authority (FDA) of Ghana to increase public awareness about ADR reporting can, therefore, be viewed as a step in the right direction to tackle low reporting of ADR in the country. It launched the Patient Engagement in Medicine Safety programme in June 2016 to enable patients to report adverse events of their medicines through community pharmacies designated as Patient Safety Centres.

In the present study, there were no significant differences in age and years of practice between pharmacists who reported ADRs and non-reporters. This was consistent with findings from other studies conducted among Portuguese pharmacists which found no statistically significant association between age as well as years of practice and ADR reporting.^6^ This was also true for pharmacists in Qatar.^17^ Community pharmacists who failed to report ADRs they had encountered mostly blamed it on a lack of reporting forms, considering the reaction as “normal” and uncertainty about the causal relationship between the drug and the suspected ADR. Though the FDA had instituted an electronic system of reporting ADR alongside the option of reporting via ADR forms (paper copy), the electronic system of reporting had not yet gained widespread prominence at the time this study was conducted, hence our focus on the paper form of reporting. Nonetheless, there was an opportunity to respond other than or in addition to what was pre-coded in the questionnaire. Pharmacists, through Pharmacovigilance workshops had also been made aware by the FDA that they do not necessarily have to establish a causal link between a suspected ADR and a drug before going ahead to report it.

According to Prakasam and colleagues, most community pharmacists in India felt that the reaction they had encountered was simple and non-serious, hence their failure to report^7^. There were similar findings among 1,001 community pharmacists in Korea with the main barriers to ADR reporting being the perception of ADR being “unserious” (77.8%) and already “well known” (81.4%), as well as uncertainty about causality (73.2%).^8^

About 87% of pharmacists who had reported an ADR within the past year had received previous training on Pharmacovigilance and ADR reporting. However, training received was not significantly associated with the reporting of ADRs when factors such as knowledge and marital status were adjusted for (AOR=2.18, 95% CI: p=0.378). Similarly, there was no significant association between Portuguese pharmacists who reported adverse reactions and those who did not concerning training received in Pharmacovigilance (X^2^=3.5, p= 0.062).^6^

In contrast, training significantly improved the ADR reporting rate among medical doctors in the Greater Accra Region of Ghana (X^2^=11.6, p <0.001).^11^ To improve ADR reporting by pharmacists and ADR reporting systems, new educational measures such as hands-on involvement with real cases have been suggested, thus placing ADR reporting closer to the daily routine activities of community pharmacists.^6^

The leading factors perceived by pharmacists in the study as contributing to underreporting of ADRs were the unavailability of the reporting forms and ignorance about the reporting procedure and where to obtain ADR reporting forms. This observation is consistent with findings among 310 health professionals in Saudi Arabia where 67.0% of respondents believed the ADR reporting forms were not widely available; 65.0% of the respondents felt that insufficient clinical knowledge contributed to underreporting while 63.0% blamed a lack of knowledge about the reporting address.^18^ Lack of awareness on how to report ADRs and concerns that the report submitted may be wrong were the two prominent perceived factors leading to underreporting among community pharmacists in Oman.^19^

By way of motivation, a little less than one-half of all the pharmacists in the current survey felt that a letter of acknowledgment per the report submitted was an adequate means of encouraging reporting of adverse drug reactions by community pharmacists. A little more than half of the respondents held the view that making ADR reporting mandatory in Ghana will improve reporting rates. Likewise, more than one-half of health professionals in Saudi Arabia stated that reporting of adverse drug reactions ought to be compulsory.^18^ In Cyprus, an even greater proportion of pharmacists supported mandatory ADR reporting.^15^

While most community pharmacists felt that reporting of ADRs is an integral part of their professional responsibility, they suggested special training in pharmacovigilance and ADR reporting to effectively carry out this responsibility. We recommend that the ADR reporting forms should be made readily available in all community pharmacies across the nation. Also, the National Pharmacovigilance Centre should consider innovative ways of reporting ADRs like using Social Media and widely publicizing direct online reporting.

Finally, further studies among other health professionals like nurses, medical assistants, herbal practitioners as well as patients to find ways of involving all these groups in pharmacovigilance activities.

Most pharmacists felt that continuous professional development and training on pharmacovigilance was an effective strategy to improve ADR reporting. This is consistent with the results of a Ugandan study where 667 out of 1589 healthcare professionals advocated for sensitization, training, and continuous education on pharmacovigilance.^20^ Moreover, about 90.0% of pharmacists in Cyprus also suggested that training in ADR reporting will help to improve the reporting rate.^15^

## Conclusion

The prevalence of adverse drug reactions (ADR) reporting among community pharmacists in the Greater Accra was 16.0%. The majority of the community pharmacists attributed their failure of reporting ADR to the unavailability of the ADR reporting forms in the community pharmacies.
